# The Unique Challenge of Coronary Artery Disease in Adult Patients with Congenital Heart Disease

**DOI:** 10.3390/jcm13226839

**Published:** 2024-11-14

**Authors:** Nunzia Borrelli, Assunta Merola, Rosaria Barracano, Michela Palma, Ippolita Altobelli, Massimiliana Abbate, Giovanni Papaccioli, Giovanni Domenico Ciriello, Carmen Liguori, Davide Sorice, Lorenzo De Luca, Giancarlo Scognamiglio, Berardo Sarubbi

**Affiliations:** Adult Congenital Heart Disease and Familiar Arrhythmias Unit, Monaldi Hospital, 80131 Naples, Italy; nunzia.borrelli@ospedalideicolli.it (N.B.);

**Keywords:** congenital heart disease, coronary artery disease, atherosclerosis, cardiovascular risk factors

## Abstract

Advances in medical and surgical interventions have resulted in a steady increase in the number of patients with congenital heart disease (CHD) reaching adult age. Unfortunately, this ever-growing population faces an added challenge: an increased risk of acquiring coronary artery disease. This review provides insight into the complex interactions between coronary artery disease and CHD in adults. We describe the peculiar features of cardiac anatomy in these patients, the possible role cardiac sequelae may play in an increased risk of myocardial ischemia, and the diagnostic challenges in this patient group. Furthermore, this review outlines the risk factors and potential mechanisms of accelerated atherosclerosis in adults with CHD by pointing out areas where current knowledge is incomplete and highlighting areas for further research. The review concludes by examining potential management strategies for this particular population, emphasizing the necessity for a multidisciplinary approach. Understanding the unique coronary risks that adults with CHD experience can enhance patient care and improve long-term results.

## 1. Introduction

Significant advancements in pediatric cardiology and surgical interventions have led to a significant increase in the number of adults living with congenital heart disease (CHD). As these individuals age, the risk of developing coronary heart disease (CAD) becomes increasingly prevalent [1].

Growing evidence suggests that adults with CHD (ACHD) are at an elevated risk of developing acquired cardiovascular diseases [2], with a 1.6-fold higher risk of developing myocardial infarction compared to the general population [hazard ratio (HR): 1.6, 95% confidence interval (CI): 1.5–1.7, *p* < 0.001] [3]. Evidence also shows a CAD rate of 5.8–7.8 per 1000 patient-years at the age of 70 years in women and men, respectively, and a higher risk in patients with severe CHD compared to mild and moderate CHD [4]. However, unlike traditional CAD, where cardiovascular risk factors (CVRFs) such as hypertension, diabetes, dyslipidemia, and tobacco use are the primary contributors, patients with ACHD face a unique additional set of predisposing factors beyond those commonly identified in the general population.

Underlying cardiac anomalies, surgical interventions, and altered hemodynamics [5] create a complex interplay that may predispose individuals to early and accelerated atherosclerosis. Moreover, the presence of coexisting conditions, such as heart failure and arrhythmias, further exacerbates vulnerability to CAD, thus compounding the clinical presentation.

Understanding the unique mechanisms by which CAD develops in patients with ACHD is crucial for optimizing prevention and management strategies. This review will examine the specific risk factors, pathophysiological processes, and clinical implications of CAD in patients with ACHD, aiming to improve the long-term outcomes of this vulnerable population of patients.

## 2. CHD and Potential CAD Risk

### 2.1. Coarctation of Aorta

#### 2.1.1. Introduction

Coarctation of the aorta (CoA) is defined as a localized or long hypoplastic narrowing of the aortic lumen, generally at the level of the ductus arteriosus insertion [5]. CoA accounts for 5–8% of all CHDs, with late adult presentation in only 10.3% of all patients with CoA [6].

#### 2.1.2. Risk Factors

Despite successful correction of the lumen obstruction, patients with CoA present an increased risk of premature mortality because of cerebrovascular accident, aortic dissection, and CAD. These adverse outcomes are not primarily influenced by the extent of the original narrowing, the chosen treatment method, the recurrence of the narrowing, or the use of artificial grafts [7]. In two independent studies, Roifman et al. [8] and Egbe et al. [9] demonstrated that CAD was primarily determined by the presence of atherosclerotic cardiovascular disease risk factors, rather than the diagnosis of CoA per se. Consequently, CAD is not an inevitable complication of CoA, but rather a preventable condition, which can be anticipated by identifying and aggressively treating modifiable cardiovascular risk factors.

Arterial hypertension, a major risk factor for atherosclerosis, is prevalent among patients with CoA [10]. Masked or uncontrolled hypertension was found in 45% of children and adolescents successfully treated for CoA [11], and this percentage is even larger in middle-aged patients with CoA [10]. Arterial hypertension provokes the atherosclerotic process through endothelial dysfunction, increased intima/medial thickness, and decreased arterial distensibility. However, persistent endothelial dysfunction and impaired arterial reactivity are also commonly found in normotensive patients after successful repair [7,12].

#### 2.1.3. Management

Regular ambulatory blood pressure (BP) monitoring is recommended for all patients with CoA to detect and manage arterial hypertension. While resting BP measurements may not accurately reflect systemic hypertension, 24 h ambulatory BP monitoring is a valuable tool for diagnosing masked hypertension. Additionally, up to 80% of patients with CoA exhibit a hypertensive response to exercise [13]. The specific morphology of the aortic arch, particularly a gothic arch, has also been linked to a higher risk of developing systemic arterial hypertension [14].

All patients with CoA should be clinically evaluated at least every year [15]. Strict management of risk factors should be assured to minimize the risk and prevent the progression of CAD. Systemic hypertension should be treated following the current guidelines [16]. According to the 2018 AHA/ACC Guideline for the Management of Adults with Congenital Heart Disease, asymptomatic patients with CoA should undergo exercise testing at least every three years. Cardiovascular magnetic resonance imaging (CMR) or computed tomography angiography (CTA) is recommended at least every 3–5 years in asymptomatic patients to assess aortic arch size and anatomy [13].

### 2.2. Turner Syndrome

#### 2.2.1. Introduction

Turner syndrome [TS] is the only surviving monomer syndrome and affects exclusively women. It is caused by either the complete or partial loss of one X chromosome in some or all somatic cells and occurs in 1 in 2000 to 1 in 4000 live-born girls [17].

#### 2.2.2. Risk Factors

Nearly half of the patients have been identified with cardiovascular anomalies, most commonly CoA and bicuspid aortic valve, mitral valve prolapse, or conduction abnormalities. However, acquired heart disease has been recognized as an increasing cause of mortality in patients with TS, accounting for 41% of all-cause mortality [18,19,20]. In particular, CAD is highly prevalent, with calcified coronary plaques recognized 63% more often (odds ratio: 1.63, 95% CI: 1.02, 2.61, *p* = 0.04) and with earlier onset than the general population [21]. The prevalence of several CVRFs has been recognized to predispose patients with TS to atherosclerosis and CAD.

Arterial hypertension is nearly three times more frequent in females with TS than in the general population [22]. This condition can occur as early as childhood, affecting 20–40% of children with TS, and may persist in up to 60% of women with TS in adulthood. After the repair of CoA, hypertension may persist even in the absence of any residual isthmic obstruction. Several factors have been recognized as responsible for arterial hypertension in TS, including obesity, metabolic disorders, estrogen deficiency, renal abnormalities, and abnormal vascular wall resistance [23].

Moreover, type 2 diabetes has been diagnosed two to four times more frequently in women with TS, and insulin resistance has been reported to occur as early as childhood in patients with TS [24]. Although there is no specific association between dyslipidemia and TS, obesity, diabetes, and inadequate estrogen replacement may cause many patients to have an unhealthy lipid profile [23]. Besides metabolic disorders, coronary anomalies may play a role in the development of CAD. Studies have shown that coronary artery anomalies are more common in the population with TS, occurring in 20% of patients compared to nearly 5% in the general population. The left main coronary artery is frequently affected, with its absence being the most reported abnormality [25].

#### 2.2.3. Management

The use of imaging techniques, such as CMR, CTA, and coronary angiography (CA), and the implementation of appropriate preventive measures and treatment strategies can help reduce the risk of CAD in patients with TS. A multidisciplinary approach is paramount, especially between cardiologists and endocrinologists, to define hypolipidemic and hypoglycemic therapy as well as specific replacement therapy. While patients with TS should be regularly evaluated for arterial hypertension throughout their lives, β-blockers or angiotensin receptor blockers should be the preferred antihypertensive drugs for their ability to reduce the growth rate of the aorta [23].

### 2.3. Transposition of Great Arteries

#### 2.3.1. Introduction

Transposition of the great arteries (TGA) is a complex congenital cardiac malformation characterized by atrioventricular concordance and ventriculoarterial discordance. Historically, the primary surgical approach for TGA was the atrial switch operation, such as the Senning or Mustard procedure. However, since the 1970s, the arterial switch operation (ASO) has become the preferred surgical treatment, involving reimplantation of the coronary arteries [26].

#### 2.3.2. Risk Factors

Patients with TGA are at a heightened risk of CAD for several reasons. The main factor is the presence of anomalies in the origin of coronary arteries, which are found in approximately 33.7% of cases [27]. Studies by Khairy et al. and Nguyen et al. have identified two coronary anatomies that are most frequently associated with adverse cardiovascular events. These are the presence of a single right coronary artery and an intramural coronary segment of the left coronary artery (type E and F sec. Yacoub) [28,29,30]. The latter is also a significant cause of reintervention in patients following ASO, ranking second in frequency only to aortic root and aortic valve procedures [31].

Secondly, the process of reimplanting the coronary arteries during ASO introduces additional risks. The manipulation and reattachment of these vessels can lead to complications such as increased risk of tension, torsion, and kinking, impairing blood flow. Therefore, coronary artery obstruction has been reported in 6.8% to 11.3% of patients with TGA after ASO, and this is usually due to mechanical obstruction of the vessels [32,33]. Additionally, Possner et al. highlight that manipulation of the coronary arteries during ASO results in sympathetic denervation, significantly impairing the maximal dilator capacity of the coronary microvasculature in young adults compared to healthy controls [34]. Finally, the risk of CAD in patients with TGA also increases with their average age of survival, as observed in other adult patients with CHD. However, there is scant or no information on the prevalence of atherosclerosis in these patients. Beyond these issues, it is also important to note that there are several reports of asymptomatic obstructive stenosis [35,36,37,38,39], which may lead to sudden adverse cardiac events later in follow-up.

#### 2.3.3. Management

As part of our center’s screening program, it is essential to perform at least one echocardiogram annually for these patients, assuming there are no complications. A benchmark assessment of the anatomic course and patency of the coronary arteries is prudent in adults from whom this information has not been obtained, primarily through computed tomography coronary angiography (CTCA). In accordance with the 2018 AHA/ACC Guidelines for the Management of Adults with Congenital Heart Disease [10], serial anatomic imaging is justified in asymptomatic individuals, with a recommendation to perform CTCA every five years as part of the screening process. CMR coronary angiography may also be considered an alternative for evaluating coronary patency. Lastly, in symptomatic patients, it is recommended to perform an inducible ischemia test, and if positive, coronary angiography at specialized ACHD centers remains essential.

### 2.4. Abnormal Variation in Coronary Anatomy

#### 2.4.1. Introduction

Congenital variations in coronary artery anatomy are rare in the general population, with a reported prevalence in the literature between 0.21% and 5.79% [40]. However, these anomalies are probably more frequent than previously estimated [41] since most cases are clinically silent and usually incidental findings in imaging exams performed with a different indication [42,43,44]. In patients with CHD, the frequency of coronary artery anomalies (CAAs) is higher, between 8.16 and 9% [45,46], representing a significant factor in surgical management and long-term prognosis. CAAs include anomalies in the origin, course, and termination of one or more coronary vessels (Table 1) [47].

#### 2.4.2. Risk Factors

While some CAAs are intrinsically responsible for cardiac ischemia due to inadequate myocardial perfusion (i.e., anomalous origin of a coronary artery from the pulmonary artery, congenital atresia, fistulas) or are related to a higher risk of sudden death (i.e., anomalous origin of the left coronary artery from the right aortic sinus with an intramural course [48]), little evidence is available regarding the correlation between CAAs and atherosclerosis.

An Italian retrospective angiography study in 2004 compared the characteristics of 22 patients with “benign” CAAs (variations not usually correlated with sudden death) and CAD, and 17 patients with CAAs without CAD. The results showed that the first group had more cardiovascular risk factors, which led to a higher rate of cardiovascular events and worse survival. The authors concluded that CAAs alone are not responsible for accelerated atherosclerosis [49].

A more recent study found atherosclerosis rates of 50% or higher in CAAs compared to normal coronary anatomy, as seen in CTCA imaging [50]. In this study, cardiovascular risk factors did not differ between the two groups, and all anomalous coronary artery anatomies examined had comparable atherosclerotic disease rates. This study confirmed previous results from the angiographic series, where the anomalous vessels showed a higher degree of atherosclerotic involvement than normal coronary arteries [51]. Some authors found that the anomalous origin of the circumflex coronary artery from the right coronary sinus or the right coronary artery carries a higher atherosclerotic burden [52,53,54].

The proposed mechanism for accelerated atherosclerosis in CAAs is the regional variation in shear stress: acute origin angles of the coronary artery, tortuous course, or turbulent blood flow could alter the endothelial integrity and predispose to vascular damage [50].

CAD in vessels with a myocardial bridge is another controversial topic. This anomaly more commonly affects the left anterior descendent artery (LAD). Some studies suggest a protective role of the bridging on the involved coronary artery [55,56]; in other case series, the atherosclerotic plaques were more common in the segment proximal to the bridging [57]. The possible cause of the endothelial damage could be the turbulence in the proximal blood flow caused by compression of the tunneled portion of the vessel [58].

Ultimately, the relationship between CAAs and CAD remains a topic of debate. Most available data are from case series, often with a limited number of patients with CAAs, and conducted in single centers.

#### 2.4.3. Management

Diagnosis of CAAs can be led by symptoms or incidental, the latter being the most frequent occurrence. Multimodality imaging provides a more accurate definition of coronary anatomy and possible atherosclerotic lesions [59]. Echocardiography, however useful as a screening tool in identifying the suspected anomalous origin of a coronary artery, requires confirmation by a second diagnostic method. CTCA and CMR, with the possibility of three-dimensional reconstructions, have replaced CA as the diagnostic gold standard for CAAs. CA, however, offers additional information on intraluminal and plaque characterization and functional assessment by fractional flow reverse, allowing percutaneous treatment when needed. Functional tests define the clinical significance of CAAs and guide therapy. A conventional stress test is often the first-line assessment of CCA symptoms and ECG modifications, despite its low sensitivity. Stress echocardiography, exercise scintigraphy, and stress cardiac MR are more accurate tests that could be offered as second-level exams in case of uncertainty [41].

### 2.5. Cyanotic Patients

#### 2.5.1. Introduction

There are conflicting opinions regarding CAD risk in cyanotic patients. Some studies suggest that cyanotic patients may be protected against CAD. In a Toronto review, only 1% (149/12,124) of patients with CHD had CAD, of whom only 5% had Eisenmenger physiology [60]. Similarly, Fyfe et al. showed there was no evidence of atherosclerosis in 59 coronary arteriograms and five necropsy specimens of patients with cyanotic ACHD (25 women with a mean age of 43 ± 3 and 24 men with a mean of 41 ± 4) [61]. In another review of CA undertaken in a CHD cohort (including Eisenmenger syndrome), no patient with cyanosis had significant CAD [2].

#### 2.5.2. Contributing Factors

The factors contributing to this lower risk may include decreased prevalence of traditional risk factors such as hypercholesterolemia, obesity, and hyperglycemia [62], as well as hypoxemia, upregulation of nitric oxide, hyperbilirubinemia, and low platelet counts [63]. Moreover, mean carotid intimal medial thickness (IMT) is significantly decreased compared with age-matched controls [64]. Another potential protective factor is the dilatation of the extramural coronary arteries due to both endothelial vasodilator effects of nitric oxide and prostaglandins released in response to increased endothelial shear stress of the viscous erythrocytosis perfusate and medial structural abnormalities (loss of medial smooth muscle and deposition of collagen). Coronary vascular resistance and flow reserve are preserved from the remodeling of the intramyocardial coronary microcirculation mediated by vascular endothelial growth factor, upregulation of vascular endothelial growth factor receptor-1, and nitric oxide [63].

Although the current evidence suggests that cyanotic patients may have a lower incidence of CAD compared to non-cyanotic patients, they are not completely protected from developing obstructive ischemic heart disease. Indeed, systemic vascular endothelial dysfunction has been documented in patients with cyanotic CHD, suggesting that they may still be at risk for atherosclerosis [65,66]. Patients with Eisenmenger syndrome are not immune to developing obstructive ischemic heart disease, although it seems to be rare [67]. In another group of patients with CHD at risk for cyanosis—patients with Tetralogy of Fallot (TOF)—CAD was more prevalent in the middle-aged and older patients with TOF, with a prevalence of 21% [68]. In another study, the prevalence of CAD in patients with TOF was 19% and 15% for mild and significant CAD, respectively [69]. Considering the high prevalence of CAD in the population with TOF, cardiovascular risk factor screening should not differ from such screening in the general population, and pharmacologic and non-pharmacologic strategies are necessary to modify these risk factors.

As survival improves in cyanotic patients, attention to conventional CVRFs becomes increasingly important to optimize outcomes and prevent cardiovascular complications.

## 3. Risk Factors and Potential for Accelerated Atherosclerosis

In the aging population with ACHD with abnormal anatomy, function, and physiology, the effect of traditional CVRFs may well be amplified since these patients often present an increased risk for heart failure and arrhythmia. Up to 80% of patients with ACHD show at least one modifiable CVRF, with overweight/obesity and systemic hypertension being the most prevalent. Thus, screening, identification, and treatment of traditional CVRFs is essential in the standard follow-up for ACHD [70].

On the other hand, in patients with complex CHD, factors different from those commonly recognized in the general population (i.e., congenital coronary artery anomalies) seem to play a pivotal role in the genesis of CAD.

### 3.1. Obesity and Overweight

Obesity and overweight are frequently present in patients with ACHD, with a prevalence similar to that in the general population [71]. Whether factors different from those recognized in the general population increase the risk of developing obesity in this cohort is unknown. The higher prevalence of obesity/overweight in patients with ACHD may result from the interplay of multiple factors. During infancy, parental overprotection with overfeeding, representing a false sense of better health in worried parents, and historical recommendations of exercise restriction potentially contribute to a low level of physical activity and an increased risk of obesity [72]. Education regarding appropriate healthy lifestyles is often inadequate; an unhealthy diet with excessive ultra-processed foods and a sedentary lifestyle represents an early threat. Moreover, patients’ uncertainty regarding the safety or benefit of regular physical exercise represents a significant barrier to physical activity [73]. Overweight and obesity may lead to a higher prevalence and severity of hypertension, hyperlipidemia, and diabetes.

### 3.2. Smoking

Smoking rates are overall lower compared to the general population, increasing in those with known CAD and in functionally limited patients for whom smoking seems to be a coping mechanism [74]. Smoking increases the risk of cardiovascular events through various mechanisms, such as reduced high-density lipoprotein cholesterol, increased platelet aggregability, and reduced carboxyhemoglobinemia [75].

### 3.3. Systemic Hypertension

According to current guidelines, systemic hypertension is defined as office systolic BP values of 140 mmHg or higher, and/or diastolic BP values of 90 mmHg or higher. A European Consensus Panel on hypertension in children and adolescents recently agreed that, for 16-year-old adolescents or older, values of ≥130/85 mmHg are adequate to diagnose systemic hypertension in this specific cohort. The prevalence of systemic hypertension is reported at 19–35% in patients with ACHD, rising to 55% in patients with known CAD. Coarctation of the aorta represents a well-known risk factor for developing systemic hypertension. Factors that may contribute to the multifactorial genesis of hypertension include arterial stiffness, altered endothelial function, renal disease (especially in cyanotic patients), concomitant endocrine dysfunction, and sleep apnea [76].

### 3.4. Diabetes Mellitus

The population with ACHD has a higher prevalence of diabetes mellitus compared to the general population. Beyond traditional risk factors, cyanosis seems to be an independent risk factor for diabetes in patients with ACHD, probably caused by the negative impact of hypoxia on glucose metabolism [77].

### 3.5. Dyslipidemia

The existing data on the prevalence of dyslipidemia in patients with ACHD are scarce, with the majority of studies demonstrating an overall prevalence similar to that in the general adult population. However, the impact of dyslipidemia on ACHD is often underestimated [78].

Wu et al. [79] demonstrated that dyslipidemia is highly prevalent in patients with ACHD, occurring in nearly one-half of their cohort. As for the general population, patients with ACHD with dyslipidemia were more likely to be obese than those without dyslipidemia. Low high-density lipoprotein cholesterol levels were most common and predominated in those with complex CHD, whereas high total cholesterol levels were often present in patients with simple and moderate CHD.

### 3.6. Accelerated Atherosclerosis in Adult Congenital Heart Disease

Accelerated atherosclerosis and acute cardiovascular events in ACHD are influenced by a complex interplay of factors. These may include abnormal coronary artery anatomy, inflammation, scarring of the coronary arteries from surgical manipulation or the abnormal anatomy underlying CHD, reperfusion injuries during cardiac surgery, ventricular hypertrophy or dilation, aortopathy with dilated aorta and increased aortic stiffness, and associated genetic syndromes [2,80,81].

In patients with CHD, accelerated atherosclerosis may be furthered by a unique anatomy. The origins of the coronary arteries may be abnormal, with aberrant courses in some cases, providing turbulence and increased shear stress areas that further promote plaque formation.

Chronic inflammation may occur in patients with CHD due to the CHD itself, the surgery for repair, or because of a genetic condition [82]. This chronic inflammatory state can promote the formation and progression of atherosclerotic plaques.

The presence of scar tissue from previous surgical interventions can disrupt blood flow and promote plaque formation. Reperfusion injuries during cardiac surgery can also contribute to inflammation and accelerate atherosclerosis.

## 4. Possible Management Option

**Figure 1:**
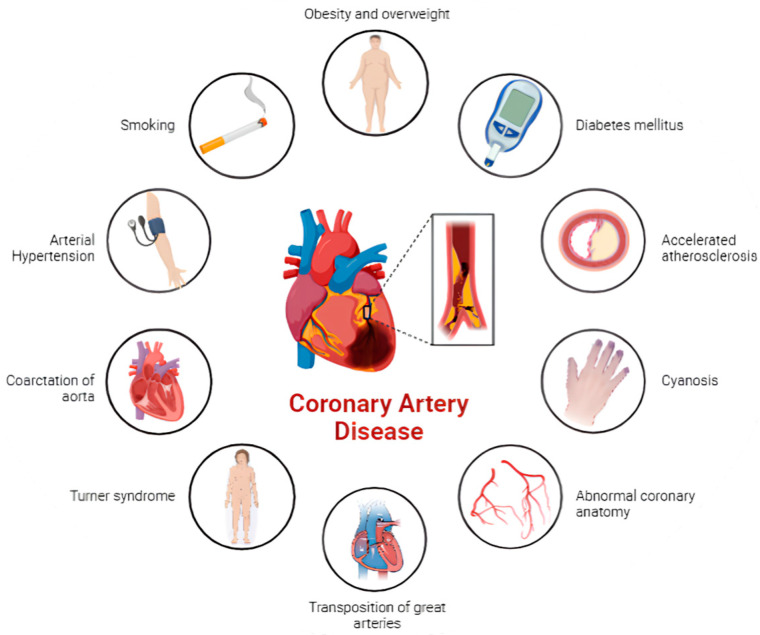
Coronary artery disease determinants in patients with congenital heart disease.

As a result of advances in the diagnosis and treatment of CHD, an increased proportion of adult patients with CHD has emerged [1]. Although these patients may experience CVRFs as do individuals without CHD, several studies have shown that they present a higher risk of developing CAD compared to the general population [2,3]. The exact processes underlying the association between CHD and the elevated risk of CAD are multifactorial and remain not completely understood. However, numerous potential contributors have been recognized. These range from an intrinsic risk carried by certain CHD and cardiac surgical repairs to the higher prevalence of endothelial dysfunction and atherosclerosis in adults with CHD. Furthermore, patients with ACHD frequently exhibit a greater prevalence of conventional cardiovascular risk factors, which may be due to genetic predisposition or lifestyle choices impacted by their condition [81] (Figure 1).

Personalized risk factor management is of paramount importance. A one-size-fits-all approach does not appear appropriate as a preventive strategy for this variegated population. On the contrary, a multidisciplinary approach appears crucial to ensure comprehensive and effective patient care. This collaborative model would involve different healthcare figures, including cardiologists, endocrinologists, geneticists, and pulmonologists. While cardiologists would play a central role in diagnosing, monitoring, and managing CAD, an endocrinologist would be crucial in addressing metabolic disorders such as diabetes and thyroid dysfunction, genetic counseling would be paramount in defining genetic cardiovascular predispositions, and a pulmonologist would manage conditions such as pulmonary vascular disease. Additionally, telemedicine is increasingly becoming more widespread in improving patient care by leveraging technology and overcoming geographic barriers. Remote monitoring of vital signs, such as BP, heart rate, and oxygen saturation, could be beneficial for treating patients with ACHD and CAD, enabling early detection of complications and timely intervention [1].

As the burden of established risk factors increases, aggressive therapeutic targets are essential, while therapeutic approaches might need to be tailored to the particular CHD lesion and any associated hemodynamic abnormalities [83].

New drug therapies seem to have potential benefits for ACHD. In light of the low adherence to the treatment of patients with ACHD and considering the importance of controlling CAD risk factors, subcutaneous administration of therapies could play a favorable role in this context. Alirocumab, evolocumab, and inclisiran have widespread use in the adult population, mainly due to their effect on PCSK9 in controlling LDL cholesterol. However, no study has been exclusively directed at their use in the population with ACHD, and specific guidelines on their use in this population are generally lacking. The possibility of a few subcutaneous injections, without additional oral medications, along with the lipid-lowering effect of these drugs, may enhance therapy adherence and improve lipid control. Weight reduction efforts should focus on overweight and obese individuals to lower the risk of CAD and improve their cardiovascular risk profile. Therefore, in such a scenario, glucagon-like peptide-1 receptor agonist administration might be extended to patients with ACHD, in whom both weight reduction and favorable effects on glucose metabolism would be of interest. However, scientific evidence of their potential has not yet been evaluated in the population with ACHD. Inhibitors of sodium-glucose cotransporter 2 (SGLT2) have shown cardio-renal benefits in several clinical scenarios, particularly in patients with heart failure [84]. Their application in myocardial infarction is supported by experimental research in animal models, showing a reduction in unfavorable cardiac remodeling and infarct size [85,86,87]. Recently, three clinical trials [88,89,90] have reported encouraging results in this setting. Further investigations may clarify the role of SGLT2 in patients with CAD and further explore their role in the population with ACHD.

## 5. Conclusions

Patients with ACHD face a heightened risk of developing CAD. Optimizing long-term health outcomes for this growing population will require a multi-pronged approach that includes early CVRF identification and management and possibly tailored CAD screening and treatment strategies. Further research is needed to refine our understanding of the underlying mechanisms and to define optimal strategies for preventing and treating CAD in patients with ACHD.

## 6. Gaps in Evidence and Future Directions

Despite significant progress, several key areas remain under-researched.

- Pathophysiological Mechanisms

A deeper understanding of the specific mechanisms by which specific CHD or surgical treatments and complications predispose individuals to accelerated atherosclerosis is needed. Studies investigating the role of inflammation, oxidative stress, endothelial dysfunction, and their long-term impact on patients with ACHD are warranted to identify potential risk factors and develop specific preventive strategies.

- Risk Stratification and Early Detection

Due to the additional risk imposed by underlying CHD, traditional risk stratification tools may not apply to this population. Several scores have been developed and validated in the general population, including SCORE2 and SCORE2-OP [91,92]. However, these scores have not been validated for the population with ACHD. Further investigation into specific risk stratification tools that consider the severity of underlying CHD lesions may help guide targeted preventive strategies.

Optimal imaging modalities for early detection of CAD in ACHD are still uncertain. New noninvasive imaging techniques tailored for patients with complex anatomy and a history of prior surgeries are needed, as is optimizing the timing and frequency of imaging screening.

Leveraging telemedicine and digital health technologies can improve access to specialized care, facilitate remote monitoring, and enhance patient engagement [1]. However, further research is needed to evaluate the effectiveness of these technologies in managing CAD in ACHD.

- Treatment Strategies

Whereas treatment strategies may need to be tailored to the CHD lesion and the presence of coronary anomalies and prior surgical interventions, further studies are necessary to determine which revascularization modality yields the best results in this population.

Some CHDs are associated with unique risk factors that may differently predispose individuals to CAD, necessitating distinct prevention and treatment approaches. For example, lipid level cutoffs are typically adapted from the general population. It is, however, still uncertain whether the goals generally accepted in the general population for LDL cholesterol and triglycerides confer similar benefits to patients with ACHD or whether specific targets need to be developed.

## Figures and Tables

**Table 1:** Classification of coronary artery anomalies.

**1. Anomalous Origin**		
a. From the pulmonary artery	b. From the aorta	c. Congenital atresia/hypoplasia
	From a different coronary sinus	
	Single coronary artery	
	High take-off from ascending aorta	
	From another portion of the aorta or other arteries	
**2. Anomalous Course**		
a. Coronary bridging	b. Aneurysm	c. Split coronary artery
**3. Anomalous Termination**		
	a. Fistula	
